#  On the Limitations of Biological Knowledge

**DOI:** 10.2174/138920212803251445

**Published:** 2012-11

**Authors:** Edward R Dougherty, Ilya Shmulevich

**Affiliations:** 1 Department of Electrical and Computer Engineering, Texas A&M University; 2 Computational Biology Division, Translational Genomics Research Institute; 3 Institute for Systems Biology, University of Washington; 4 Department of Electrical Engineering, University of Washington; 5 Department of Bioengineering, University of Washington

**Keywords:** Complexity, Gene regulatory networks, Epistemology, Experimental design, Genomics, Knowledge discovery, Modeling, Validation.

## Abstract

Scientific knowledge is grounded in a particular epistemology and, owing to the requirements of that epistemology, possesses limitations. Some limitations are intrinsic, in the sense that they depend inherently on the nature of scientific knowledge; others are contingent, depending on the present state of knowledge, including technology. Understanding limitations facilitates scientific research because one can then recognize when one is confronted by a limitation, as opposed to simply being unable to solve a problem within the existing bounds of possibility. In the hope that the role of limiting factors can be brought more clearly into focus and discussed, we consider several sources of limitation as they apply to biological knowledge: mathematical complexity, experimental constraints, validation, knowledge discovery, and human intellectual capacity.

## INTRODUCTION

Near the end of his recent history of cancer, *The Emperor of Maladies*, and following almost 450 pages of a depressing empiricism, Siddhartha Mukherjee waxes hopeful in regard to a future medicine grounded in translational genomics:

- Gene by gene, and now pathway by pathway, we have an extraordinary glimpse into the biology of cancer. The complete maps of mutations in many tumor types (with their hills, valleys, and mountains) will soon be complete, and the core pathways that are mutated fully defined. But as the old proverb runs, there are mountains beyond mountains. Once the mutations have been identified, the mutant genes will need to be assigned functions in cellular physiology. We will need to move through a renewed cycle of knowledge that recapitulates a past cycle – from anatomy to physiology to therapeutics [1].

Mukherjee does not run from the difficulty but he certainly appears to envision a therapeutic revolution based on scientific progress that far surpasses the medicine of the recent past. 

This is a much different perspective than one taken only a decade earlier by James Le Fanu in his book, *The Rise and Fall of Modern Medicine*, when he writes:

- Medicine, like any field of endeavor, is bounded by its concerns – the treatment of disease – so success necessarily places a limit on further progress…. As of the moment, it is not clear whether or how the last challenge left – the discovery of the causes of disease like multiple sclerosis and leukemia – is indeed ‘soluble.’… The limited prospects of future medical advance should by now be well recognized [2].

Le Fanu, too, argues that the past has been dominated by empiricism, but he is not so enamored by the prospects of translational medicine. For him, the limitations of science inevitably place limits on science-based medicine and he clearly believes that the limitations on biological knowledge have already been approached to the point that they limit major medical advances. Surely, one cannot deny that limitations on scientific knowledge limit its application, so that Mukherjee’s optimism must be tempered by any limitations that exist. On the other hand, Le Fanu places stark limitations on science before science has even begun in earnest. He admits that limitations have been assumed before in physics, only to be quickly shattered. Yet somehow it is to be different with biology. 

As a modern science, biology is in its nascent years. In 1949, Norbert Wiener wrote, “Many perhaps do not realize that the present age is ready for a significant turn in the development toward far greater heights than we have ever anticipated. The point of departure may well be the recasting and unifying of the theories of control and communication in the machine and in the animal on a statistical basis” [3]. As biological science turns to its natural home in control, communication, and information, it will have open to it a vast store of systems theory gained over the last 75 years. To judge it a failure before it has hardly begun would be akin to arguing that humans cannot walk on the moon before the arrival of Newton. Whereas a scientific pessimist of the Sixteenth Century did not possess almost a century of the calculus that would spur the advancement of a new physics and translate into space travel, today we possess three-quarters of a century of the mathematical theory that will propel a new biology that will translate into systems medicine.

Nonetheless, there will be limitations. An understanding of these limitations is useful for research because it can serve as a guide to the kinds of problems that confront the researcher and as a prescription for the kinds of auxiliary advances that need to occur to mitigate those limitations, for instance, the kinds of experimental apparatus that need to be developed to support fundamental advances in biological knowledge. Limitations on biological knowledge arise from numerous sources, all finding their roots in the scientific epistemological triad: (1) knowledge represented by a mathematical model, (2) operational definitions to tie the model to experiments, and (3) agreement (in some defined sense) of model-based predictions and experimental outcomes. When we speak of limitations we do not necessarily refer to permanent limitations; more generally, we are concerned with impediments, many of which can be more or less overcome to expand the knowledge domain. For instance, while today certain matrix operations critical to the design of optimal therapeutic intervention are computationally intractable, there is no doubt that in twenty years the domain of tractability will be increased.

Obviously, limitations to knowledge are relative to the epistemological ground of that knowledge, in this case, the ground of scientific knowledge. It is not our intent to delve systematically into scientific epistemology, in particular, as it relates to biology. For an in depth study of the issue, we refer to [4]; for a synopsis that we believe provides sufficient background for the current paper, we refer to [5].

## MATHEMATICAL COMPLEXITY

Because scientific knowledge is constituted in a mathematical model and the full scope of a theory is manifested in propositions derived within the model, fundamental limitations arise from the structure imposed on the mathematical model by the nature of the science, in particular, its formal mathematical structure and its internal tractability, that is, the degree to which one can formulate relations within the theory. We incorporate all of these limitations under the heading of “mathematical complexity.” While it may be possible to write down a large number of equations describing behavior to a fine degree, the ability to derive closed-form analytic solutions for various aspects of the model deteriorates with increasing model complexity. Examples include deriving limit cycles and mean first passage times in Markovian models of gene regulatory networks, both of which characterize important phenotypic properties, or expressing in closed-form the steady-state distribution, which plays a central role in deriving therapeutic intervention strategies [6]. Thus, if one desires analytic representation, model constraint becomes mandatory.

More generally, owing to the vast number of quantitative variables within the cell, or any larger biological system, such as gene and protein expressions, and the involved relationships among these variables, it is impossible to write down a system incorporating more than a small portion of them. This difficulty stems not only from the sheer mass of variables and relations but also from the inability to measure such variables and their relationships due to experimental constraints imposed by measurement technologies, as discussed in the following section. This leads inevitably to model stochasticity resulting from latent variables and therefore to systems for which analytic treatment is virtually impossible, for instance, large systems of stochastic nonlinear differential equations. In general, as we decrease the number of relevant variables within the model, stochasticity increases. The size of the system is decreased at the cost of increased randomness and a consequent loss in predictability with regard to the phenomena being modeled. This is a fundamental trade-off in all modeling: model tractability versus phenomenal predictability. The problem is greatly heightened in biology in comparison to man-made systems because biological systems have much greater complexity than man-made systems, in particular, with regard to the nature of their interactions. Hence, biological systems are forced more strongly into these tradeoffs. 

To illustrate the stochastic effect of latent variables, we consider a network involving the widely studied tumor suppressor gene p53, which serves as a transcription factor for hundreds of downstream genes in mammalian genomes and has been widely studied. Expression of these downstream genes can modulate cell cycle progression, repair damaged DNA, and induce senescence and apoptosis. Fig. (**1**) shows some major pathways involving p53 that are activated in the presence of DNA double strand breaks [7]. In [8], two Boolean networks are derived in which the pathways of Fig. (**1**) are manifested. In each, states are of the form [ATM, p53, Wip1, Mdm2], with dna_dsb being the DNA damage input, which is external to the network. Depending on the value of dna_dsb, 0 (no damage) or 1 (damage), we obtain a different network. A Boolean network is a binary-valued network in which a gene value (0 or 1) is determined by logical rules involving gene values from the previous time instant [9]. In this case, two Boolean networks are determined by the following logical rules, depending on the value of dna_dsb:

$${\mathrm{ATM}}_{\mathrm{next}}=\bar{\mathrm{Wip1}}\cdot \left(\mathrm{ATM}+\mathrm{dna}\_\mathrm{dsb}\right)$$

$${\mathrm{P53}}_{\mathrm{next}}=\bar{\mathrm{Mdm2}}\cdot \left(\mathrm{ATM}+\mathrm{Wip1}\right)$$

$${\mathrm{Wip1}}_{\mathrm{next}}=\mathrm{p53}$$

$${\mathrm{Mdm2}}_{\mathrm{next}}=\bar{\mathrm{ATM}}\cdot \left(\mathrm{p53}+\mathrm{Wip1}\right)$$

Here, the symbols ·, +, and ¯ represent logical conjunction, disjunction, and negation, respectively. The state transition diagrams for these are shown in Fig. (**2**): (**a**) dna_dsb = 0; (**b**) dna_dab = 1. Absent damage, the network evolves into a single attractor state (0000); with damage, the network evolves into an attractor cycle in which p53 may be expressed or unexpressed. If one were to observe the network without knowing the damage status, then network behavior would appear stochastic, for instance, 0001 → 0000 when dna_dsb = 0 and 0001 → 1000 when dna_dsb = 1. A “probabilistic Boolean network” (PBN) results from considering the two Boolean networks as “contexts” of a single network governed by one of the Boolean networks at any given time point, where there is a probability of switching the governing network [6]. In this simple case, there is only one variable external to the network leading to stochasticity; in fact, there can be many such variables.

The basic point regarding complexity is that it leads inexorably to compression: models must be reduced to make them tractable. While the general concept may seem straightforward, what is not straightforward is characterizing the relation of compression to specific functionality. Compression increases uncertainty, but it may not unduly affect one’s aims for the system. For instance, if those aims are medical and the compression does not involve the regulatory mechanisms upon which the aims depend, then the compression is of no consequence. This means that compression (equivalently, model constraint) should be done with one’s ends in mind. If one begins with a complex model, say a gene regulatory network with 20 genes, and wishes to reduce it to a network with 12 genes in order to facilitate derivation of a control policy (therapeutic regimen), then that compression should be done so as to maintain, to the extent possible, the information required for such derivation. In other words, compression should be goal-dependent. For example, the network in Fig. (**1**) could be expanded to include other regulators of p53, such as the checkpoint kinase 2 (CHK2), which like ATM, activates p53 and is inhibited by Wip1, in addition to being activated by ATM itself. Although it is known that CHK2 is important for shaping the dynamics of p53 [10] and, in the Boolean network context, its inclusion would have the effect of doubling the total number of states, the reduced network (without CHK2) nonetheless captures the essential behavior of p53 under no damage and double-strand-break damage conditions.

Methods need to be developed to reduce models while preserving important information for the task at hand, such as therapeutic intervention. This requires appropriate (canonical) model representation so as to separate out unneeded structure, a classic problem in signal processing. It also requires characterization of approximation accuracy for the reduction, where approximation is related to the goal of the modeling and the reduction. A scientist might be satisfied with the original network and judge it superior to the reduced one because it provides better prediction, but the translational scientist (engineer or physician) requires the reduced network in order to accomplish the translational mission, albeit, perhaps with decreased performance than would be the case with the original network. Unfortunately, with the latter being impossible, a trade-off must be made or else one is paralyzed. 

While too much complexity presents a problem, paralysis can also ensue at the opposite end of the model reduction spectrum. This is starkly evident in genome-wide association studies (GWAS). The goal of such studies is to identify genetic variations associated with a particular disease and is based on rapidly scanning genomic markers across the genome from affected and unaffected populations [11, 12]. In a sense, such studies represent the ultimate compression to the point where 'the model' is typically reduced to a univariate statistical test, such as a chi-square test, which produces a p-value for the significance of the so-called odds ratio – the ratio of the proportions of individuals in each group (case/control) having a specific allele. Since millions of markers (single nucleotide polymorphisms, or SNPs) are tested, the p-values must be corrected for multiple testing and only very low ones are deemed to be significant. This requires very large sample sizes and recent studies are approaching 200,000 individuals [13]. 

Since for virtually all complex diseases, such as cancer or autoimmune diseases, no single marker carries sufficient explanatory power, a natural idea is to move toward higher complexity by considering combinations of markers. However, this has the effect of only exacerbating the multiple testing problem and demanding yet larger sample sizes. The burgeoning field of systems genetics is attacking this problem by introducing prior information, possibly computationally inferred from complementary data sets, about molecular networks that ultimately determine the phenotype. The use of such prior knowledge has the potential to substantially mitigate sample size requirements and increase the power of identifying genotype-phenotype relationships. Thus, from the engineering (translational) perspective, models must be reduced for tractability, but not to the point where their use becomes impractical due to other factors.

Although model reduction is typically associated with translational science, the scientist too must deal with compression. If analytic representations for important systems properties are desired so as to fully appreciate the scientific content of the system, then complexity trade-offs are often necessary. Here one usually speaks of approximations. Rather than deduce exact relations as might hold for the full system, the scientist must derive approximate solutions, which ultimately means that mathematical complexity has been reduced. For instance, one might have a differential equation model and reduce it to a discrete model or eliminate “very small” terms from infinite expansions. In either case, the solutions only hold approximately (in some sense) relative to the full system, just as do the solutions in a reduced model relative to the full model. 

While we have focused on mathematical complexity, this issue is closely related to computational complexity. Very often one must employ large calculations to derive mathematical characteristics of the system, say the steady-state distribution. In this case, mathematical intractability is really computational intractability. It is not that one cannot solve the relevant equations and write down the solution; rather, the computations involved in the solution are not feasible (although they might be in the future). For instance, if one considers a Markovian binary gene regulatory network with 40 genes, then there are 2^40^ states and the transition probability matrix is 2^40^ by 2^40^, which makes finding the steady-state distribution intractable.

We have emphasized the important and limiting role played by stochasticity in biological science, where stochasticity is incorporated in the mathematical model. There is another way in which uncertainty can appear in the modeling process. Rather than have a single model, stochastic or deterministic, one might wish to take a “robust” view of the situation and postulate an “uncertainty class” of models, the idea being that we believe the model belongs to the uncertainty class but are uncertain as to which model in the class applies in the present situation. In this case, rather than finding some optimal operation on the model, such as finding an optimal intervention strategy, one tries to find an operation that is robust across the uncertainty class, in the sense that it performs reasonably well on all models in the class. This idea was first introduced in the framework of finding system filters that depend on a covariance matrix when the exact matrix is unknown, our only knowledge being that the covariance matrix of interest belongs to an uncertainty class of covariance matrices [14]. The early work took a mini-max approach, the goal being to find the filter with best worst-case performance across the uncertainty class [15, 16]. Later, a Bayesian approach was proposed in which a probability distribution is associated with the uncertainty class [17]. The Bayesian approach was then applied to find optimal robust control strategies in the context of gene regulatory networks to achieve beneficial therapeutic intervention across the uncertainty class of networks [18]. 

## EXPERIMENTAL CONSTRAINTS

The difficulty in specifying many of the variables and their interrelationships in a model frequently stems from experimental limitations imposed by current measurement technologies. As technologies evolve and new measurement modalities come online, which has been the case over the past several decades in biology, the domain of modeling becomes expanded, entailing the mathematical, statistical, and computational difficulties associated with greater complexity. It is thus paramount to understand how experimental feasibility constrains model development.

Consider again models of genetic regulatory networks. Typically, such models represent the collective behavior of genes and their products, RNAs and proteins, which constitute highly dynamic, multivariate, and nonlinear interactions. To even begin to appreciate the enormous complexity of these molecular interactions, let us sketch out some of the regulatory mechanisms involved. 

For instance, transcription factors, such as the aforementioned p53, are proteins that bind to their cognate recognition sites encoded by specific DNA sequences and regulate the expression of other genes that contain these sequences in their *cis* regulatory regions. Transcription factors can frequently function jointly, either directly binding in complexes with other proteins or through combinatorial binding to the promoter architecture of their target genes [19], and they can have activating or inhibitory functions. The target genes of transcription factors are often other transcription factors, resulting in feedback or feedforward mechanisms that play important roles in modulating the dynamics of gene expression and responses to environmental cues [20].

Small non-coding RNAs, such as microRNAs, play similar roles in the cell, typically by suppressing the expression of other genes by means of translational repression or transcript degradation [21, 22], forming regulatory networks known to interplay with transcription factor networks. For example, microRNAs can suppress the expression of transcription factors and transcription factors can regulate the expression of microRNAs by binding to their promoters (regulatory regions) or the promoters of genes harboring the microRNAs in their intronic regions.

DNA, tightly packed in the nucleus of a eukaryotic cell and packaged into chromatin, is configured in three-dimensional space in a way that certain genomic regions are accessible to other proteins, such as transcription factors, while others are not. This process of genomic accessibility profoundly affects which genes are expressed and which are silenced, and itself is highly dynamic [23]. Certain proteins, themselves naturally encoded by genes and thus controlled by genetic regulatory mechanisms, are able to chemically modify components of chromatin, such as histones, through processes such as methylation, phosphorylation, or acetylation, and thereby dynamically alter chromatin structure and, consequently, accessibility of DNA to other proteins [24-27]. This process is called “epigenetic regulation.” Such post-translational modifications extend beyond chromatin modifiers to many other proteins, which themselves form vast networks of protein-protein interactions, themselves also dynamic. Such networks play important roles in transducing signals within the cell by propagating information from outside the cell to its nucleus [28, 29].

This description in no way attempts to be comprehensive and only superficially touches on the enormous complexity of molecular networks in a single living cell. It ignores other fundamental aspects of regulation, such as RNA binding proteins, metabolic networks and allosteric effectors, and, importantly, spatial organization of all these molecules within the cell. It is somewhat astounding that most of these behaviors and interactions can already be directly measured on a global scale, thereby lending themselves to experimental inquiry.

For example, the advent of high throughput sequencing technologies allows for global measurements of mRNAs, including their alternatively spliced isoforms, and microRNAs, through a methodology called RNAseq [30]. This is now becoming possible on a single cell level [31]. Global patterns of transcription factor binding to DNA, as well as those of histone modifiers, can be measured using ChIPseq, which combines chromatin immunoprecipitation (ChIP) with massively parallel DNA sequencing [32]. Protein expression, including post-translational modifications, can be measured with a variety of technologies, such as by the recently developed selected reaction monitoring (SRM) that uses targeted quantitative proteomics by mass spectrometry [33]. At the single cell level, a modest number of proteins can be measured by flow cytometry in a highly quantitative manner [34, 35] and, more recently, by mass cytometry (CyTOF), which promises the ability to measure hundreds of proteins per cell [36].

Despite this impressive ability to measure the abundances, states, and interactions of biomolecules on a global scale, current experimental capabilities are still woefully inadequate for constructing all but the simplest and reduced models of biomolecular networks in a cell. This is due to a number of fundamental experimental limitations. First is the issue of sensitivity. Most biological measurements are performed on cell populations including measurements of mRNAs, microRNAs, and proteins. The same is true for measurements of protein-DNA interactions using ChIPseq. This is a fundamental problem, since even nominally identical cells can exhibit great heterogeneity in the abundances of transcripts and proteins, due to numerous factors including thermal fluctuations, intrinsic stochasticity or noise in gene expression, and even minor differences in the cellular microenvironment, which can be amplified by the cell [37-39]. Thus, population level measurements only yield average behaviors, which can be highly misleading. 

Returning to the p53 signaling network, p53 expression levels, when measured in a cell population, first increase dramatically in response to double strand breaks, but then decrease in a series of damped oscillations, with the amplitude decreasing over time [40]; however, single-cell analysis using fluorescently tagged p53 reveals that individual cells exhibit undamped pulses with fixed amplitude and duration [41]. One reason for this phenomenon is that the number of cells exhibiting pulses is decreased with time and synchronization between individual cells is eventually lost [8]. Population level average measurements naturally mask this effect.

Although such single cell dynamics can be measured using fluorescently labeled constructs, such as luciferase, and quantified through automated microscopic imaging, one sacrifices the ability to measure behaviors globally. Only a handful of genes or proteins can be measured in this manner. One promising recent technology is microfluidics, which permits quantitative measurement of multiple single cells in a highly controlled microenvironment that allows parallelization and multiplexing, thereby making it possible to measure multiple genes under multiple conditions simultaneously [42-44]. Nonetheless, global and dynamic measurement of gene activity at a single cell level is still experimentally out of reach.

This is not to say that population level measurements are not useful in model building and validation. Indeed, a model constructed on a single cell level can be used to make predictions on a population level. For example, single cell models can be studied using stochastic simulation algorithms, such as the well-known Gillespie algorithm [45], and validated at a population level by measuring the distributions of protein expressions from a large number of cells using flow cytometry [46]. Naturally, statistical limitations must be carefully considered and sample sizes should be large enough to deal with heterogeneity.

A related experimental limitation stems from the inability to measure different time scales concomitantly. For example, signaling networks, through processes such as phosphorylation, protein conformational changes, and physical movement of signaling compounds by diffusion, frequently operate on a timescale of seconds or milliseconds [47], but transcriptional regulatory networks exhibit dynamics on a timescale of minutes to hours [48]. Since these processes are coupled, it becomes necessary to measure them jointly in order to construct mathematical models describing their behavior. This is currently impossible, since such measurements require different techniques that cannot be carried out on the same cells. Furthermore, these techniques are generally destructive, meaning that the cell is killed during the process of measurement. This challenge can be partially overcome by assuming timescale separation, meaning that fast interactions are assumed to complete before the slow interactions begin to change the concentrations of proteins. Mathematically, this can be achieved by steady-state approximations on the fast timescale, which are used as inputs to the slow timescale models [49].

Just as biological systems are multiscale in the temporal domain, they also exhibit multiscale characteristics in the spatial domain. Firstly, intracellular molecular dynamics are highly nonhomogeneous, with most molecular species localized to only certain subcellular compartments, such as organelles, including the nucleus or the nuclear membrane, peroxisomes, mitochondria, endoplasmic reticulum, cellular membrane, and so on. This presents problems for modeling efforts that make assumptions of internal homogeneity, including systems of ordinary differential equations describing these interactions. The cell is far from a well mixed bag of molecules. Molecular imaging techniques, employing quantum dots and other nanoparticles, can be used to track the subcellular localization of individual molecules and colocalization of multiple molecules [50, 51]; however, these techniques are currently limited to only several molecular measurements at a time.

Secondly, multicellular systems, especially higher Metazoa, are organized across multiple spatial scales. Cells communicate with each other not only through chemical signaling, by means of secretion of various diffusible factors, such as growth factors and cytokines, but also via physico-mechanical interactions as they move, adhere to each other, divide, and interact with the extracellular matrix. These inter-cellular interactions comprise tissues, typically constituted by multiple different cell types that build structures, such as blood vessels. The spatial organization further extends to organs and eventually to whole organisms. 

The feasibility of constructing molecular and cellular models is greatly constrained by the experimental capabilities of measuring such multiscale behaviors. Yet, this is necessary for attacking complex diseases such as cancer, which is inherently a multiscale phenomenon [52]. Indeed, consider that even a microtumor contains billions of cells of different types, including tissue specific cancerous cells, infiltrating innate and adaptive immune cells, endothelial cells that construct blood vessels, and other stromal cells. Thus, even though molecular disruptions to regulatory networks in cancer may be the underlying initiating events, a full understanding of cancer as a multicellular phenomenon must rely on multiscale models spanning multiple temporal and spatial scales of organization. Molecular *in vivo* imaging will need to evolve to the point where highly parallel and dynamic measurements can be made across multiple scales so as to inform construction of mathematical models for capturing these complex behaviors and designing therapeutic approaches. Until that time, simplifying assumptions concerning separation of scales and homogeneity must be made to reduce model complexity, as already discussed. Predictions from such simplified models can be tested by cleverly designed experiments that measure particular characteristics of the systems and partially validate the models. This brings us to the important topic of model validation, which carries with it its own particular limitations and challenges.

## VALIDATION

Validation depends on predictions from the model agreeing (in some statistical sense) with experimental observations. Validating every relation in a complex model is typically beyond experimental feasibility. One may lack the experimental capability to obtain the observations relevant to certain predictions or the number of experiments may simply exhaust time or resources owing to model complexity – the number of relations to check. Hence, one seeks to validate some characteristics derivable from the model, such as the connectivity or the steady-state distribution in the case of a regulatory network. Albert Einstein writes, “In order that thinking might not degenerate into ‘metaphysics,’ or into empty talk, it is only necessary that enough propositions of the conceptual system be firmly enough connected with sensory experiences” [53]. Validation is a process and validity is relative to that process. The model must be connected to observations, but the specification of this connection in a given circumstance is left open – in particular, the specification of what is “enough.” 

The characteristic (or characteristics) one chooses for validation depends on the ability to perform experiments and the aspects of the model with which one is most concerned. The latter is a pragmatic question. For instance, if one is interested mainly in the long-run behavior of a network, validating the steady-state distribution is of prime interest. Since many networks possess the same steady-state distribution, or ones very close to the steady-state distribution of the model, such an approach only validates that the model belongs to a class of networks whose steady-state distributions are concordant with the data; indeed, the transient behavior of the model may differ greatly from the observed transient behavior were one to observe transient behavior of the biological system itself. 

Although validation is pragmatic, depending on the choice of validation criteria, it is nonetheless intersubjective because the validation criteria are intersubjectively understood. Two scientists may differ on the validation criteria they wish to apply; nonetheless, each understands the other’s criteria. This is why it is important to always specify the validation criteria when proposing a model; otherwise, the model is not intersubjective, the phenomenal content of the model remains unknown, and there is no science. 

To illustrate the notion of characteristics in the context of gene regulatory networks, we consider a mammalian cell cycle network proposed in [54]. Under normal conditions, cell division coordinates with growth in a process tightly controlled via extra-cellular signals indicating whether a cell should divide or remain in a resting state. The positive signals (growth factors) instigate the activation of the key gene Cyclin D (CycD). Should p27 be mutated, abolishing its expression, it is possible for both CycD and Rb to be simultaneously inactive and, consequently, for the cell to cycle in the absence of any growth factor [55]. (Table **1**) summarizes the mutated Boolean functions for eight genes: Rb, E2F, CycE, CycA, Cdc20, Cdh1, UbcH10, and CycB. The growth factor input is external to the cell and its value is determined by the cell's environment. The expression of CycD reflects the state of the growth factor and is not part of the network. Depending on the expression status of CycD, one of two context Boolean networks is obtained, corresponding to whether CycD = 0 or CycD = 1. The PBN network model is completed by defining a probability of switching contexts and a small probability that a gene may randomly flip its value. Figs. (**3** and **4**) show the connectivity graph and steady-state distribution of the network, respectively. The steady-state distribution is defined by π(1), π(2),…, π(* m*), where *m* is the number of states, such that, no matter what state the network is currently in, the probability of being in state *j* after a very large number of transitions converges to π(*j*). For more details on this network, see [56], where structural intervention is considered to reduce the long-run probability of being in cancerous states.

In the classical deterministic environment, one makes a prediction from the model, conducts an experiment in which one of the observables corresponds to the prediction and checks for agreement. Owing to experimental variability, one has to allow for some disagreement between the predicted and observed values, but this is purely an issue of experimental accuracy and, if we were to assume perfect accuracy, the conclusion would either be agreement or disagreement. In the case of disagreement, we would reject the model – the theory would be falsified. On the other hand, agreement simply means that the model is accepted insofar as the particular prediction (characteristic) is concerned, but remains open to rejection should future predictions fail to agree with the relevant observations. The model is contingently validated.

Because biological models are inherently stochastic, one cannot hope to get agreement between prediction and observation (even discounting experimental variability). Thus, to check a prediction, one must employ a hypothesis test involving the distribution of the prediction, assuming the model. Given this “null” distribution and an observation (or set of observations), a decision is made to accept or reject the hypothesis based on some region of rejection. As in standard hypothesis testing, this “critical region” is determined via probabilities from the null distribution. We note in passing that “accepting” the null hypothesis is to be interpreted as “failing to reject” it – in the absence of evidence against it, one simply continues to assume it to be true. We note further that there is abundant criticism of this classical approach to statistics, primarily rooted in the belief held within the Bayesian philosophy that experiments should not lead to a conclusion, but rather to a probability or estimate with associated confidence intervals. There being abundant literature on this topic, e.g. [57, 58], we return to the classical approach and illustrate the ideas through examples.

Suppose one has a proposed model for a gene regulatory network involving 10 genes and considers the steady-state distribution as the characteristic to be validated. Keeping in mind Einstein’s dictum, we need to make some predictions involving the steady-state distribution. Let us suppose we have access to steady-state data, for instance, 200 gene-expression microarrays. Each array gives us one 10-gene state-vector measurement in the steady state. From these we form an observed steady-state distribution, say by simply taking the empirical distribution. We then compute a metric between the steady-state distribution of the proposed model and the observed steady-state distribution. For the sake of demonstration, we take the *L*_1_ distance between the distributions, defined by

$${\left\|\pi_{\mathrm{model}}-\pi_{\mathrm{observe}}\right\|}_{1}=\sum_{j=1}^{m}\left|\pi_{\mathrm{model}}\left(j\right)-\pi_{\mathrm{observe}}\left(j\right)\right|$$,

where *m* is the number of states. Letting *d* denote the distance, we require a value *c* based on a null distribution so that the model is accepted if *d* ≤ *c* and rejected if *d* > *c*. To construct the null distribution we generate 10,000 empirical steady-state distributions from the model, each generated by selecting 200 random points from the steady state, and then compute the *L*_1_ distance for each of the 10,000 distributions to generate a null distribution for the *L*_1_ distance, *D*. The critical value *c* for this null distribution can then be chosen so that the probability of *D* > *c*, meaning *D* > *c* given the model, is less than some agreed upon small value α. The model is then rejected or accepted based upon whether the observed *L*_1_ distance *d* exceeds or does not exceed *c*, respectively. Note the pragmatics involved: choice of characteristic (steady-state distribution), sample size (200), formation of the steady-state distribution from the data (the empirical distribution), choice of metric (*L*_1_ distance), and choice of decision criterion (*D* > *c* and α).

To further illustrate the pragmatics involved, let us consider another validation procedure for the same network. This time we use the connectivity graph, whose vertices are genes and whose edges indicate some degree of regulatory effect between the genes. Given data, in this case, time-course expression measurements, we apply some algorithm to construct a connectivity graph from the data [59, 60] and then compute the Hamming distance, *h*, between the constructed graph and the true connectivity graph for the network, the Hamming distance between graphs *G*_1_ and *G*_2_ being the number of edges in *G*_1_ not in *G*_2_ plus the number of edges in *G*_2_ not in *G*_1_. The null distribution for the Hamming distance is constructed by generating 10,000 data sets from the model, constructing 10,000 connectivity graphs from the data via the same algorithm, and then computing 10,000 Hamming distances to form the null distribution for the Hamming distance *H*. From there we proceed as in the case of the steady-state distribution, this time the critical region being *H* > *c*. Note again the pragmatics involved: characteristic (connectivity graph), sample size (200), formation of the connectivity graph from data (the chosen algorithm), the choice of metric (Hamming distance), and choice of decision criterion (*H* > *c* and α). 

If one chooses to use a number of characteristics, then the likelihood of at least one observed metric exceeding the critical value is no longer α. This is the problem of multiple comparisons and a correction factor needs to be employed. Since this is purely a statistical problem, we shall not discuss it further. 

Strictly speaking, a model should be validated based on the concordance of model-based predictions and future measurements; however, one may gain some degree of validation by checking predictions against existing experimental results in the literature. This is problematic because it is difficult to judge whether the model has been developed independently of the known results and because it is unlikely that a previous experimental protocol would be consistent with that required for testing the newly proposed model; nevertheless, absent the resources to perform a proper hypothesis test, concordance, or lack thereof, with the literature at least lets one check model feasibility. A good example of this kind occurs in [61], where a differential-equation model is constructed to characterize nutritional stress response in *E. Coli*, the model is reduced to a graphical model, and the long-run behavior of the reduced model is compared to experimental results in the literature.

We close this section by noting that we have been considering the scientific validity of a model, not the performance of an inference procedure that aims to produce a model from data. The validity of an inference procedure is evaluated relative to its ability to infer a hypothetical model from sample points generated by the model, perhaps perturbed by noise [62, 63].

## KNOWLEDGE DISCOVERY

Because biological knowledge involves stochastic non-linear systems exhibiting extensive parallelism, redundancy, and feedback, it is difficult to form conceptualizations and difficult to design experiments to aid in conceptualization. Because scientific knowledge is constituted within the framework of mathematics, one must address within that framework the twin issue of formulating biological knowledge and obtaining observations that facilitate such formulation. 

The formulation of a scientific theory presupposes prior knowledge. The creative process involves integrating prior knowledge, in the form of mathematical propositions, with observations. Data in the absence of knowledge leaves one with a virtually unbounded model space in which to configure mathematical theories. This would be like having a random proposition generator that generates models to fit the data – an enterprise that has not been entirely forsaken in the scientific community [64-66]. Even if one begins with a model structure, say the assumption that the model must consist of a system of stochastic differential equations, the model space remains exceedingly large. The scientist must come to the table with sufficient knowledge of the problem that he or she can formulate a small class of models for which it remains only necessary to utilize data to estimate some set of parameters to instantiate the model. 

Prior knowledge can be roughly divided into two types. We refer to the first as “biological organizational principles.” As the name implies, these are general principles that constrain and focus the conceptualization on feasible systems and constrain the model space for inference. Some examples of such principles in the context of network models are: connectivity constraints (e.g. distribution laws), dynamics (e.g. criticality), functionality requirements (e.g. robustness to environmental perturbations), energy efficiency, etc. A second kind of knowledge concerns existing relations among the variables of interest that constrain the model by requiring it to be consistent with these prior relations. Examples of these relations include known regulatory mechanisms among transcription factors and their target genes, protein-protein interactions, and targets of microRNAs.

In sum, the discovery process requires the discoverer to carry a large toolbox. This toolbox needs a wide array of mathematical structures within which one can conceptualize and it needs an array of biological organizational principles which the scientist can apply to model formulation. This demand puts limitations on the discovery of biological knowledge. This is particularly true in regard to the mathematical requirement. Owing to stochasticity, highly multivariate interaction, nonlinearity, distributed control, and asynchronous timing, the mathematics of biological systems is more difficult than that of most engineering disciplines. Hence, the mathematical limiting effect on the development of biological knowledge is greater than that in engineering.

While mathematical knowledge is beneficial because it provides formal structures for conceptualization, science is not simply mathematics. The scientist requires observations upon which to cogitate when conceptualizing. For the ancient and medieval scientists, observations were haphazard and useful observations were dependent on good fortune. It was Francis Bacon, in the *Novum Organum* (1620), who articulated the modern conception of experiment when he wrote, “The true method of experience…first lights the candle, and then by means of the candle shows the way; commencing as it does with experience duly ordered and digested, not bungling or erratic, and from it educing axioms, and from established axioms again new experiments” [67].

It was Galileo who first made this concept central to scientific practice. The post-Galilean scientist chooses how Nature is to be probed so as to focus attention on those aspects of Nature that correspond to the issue at hand. He or she does not approach Nature blindly in the hope that some nugget might fall from the sky. To arrive at a model, or conceptualization, the scientist begins with ideas, which one might see as preliminary conceptualizations, from which he or she designs experiments. Reflecting on Galileo and the planned experiment, Immanuel Kant made one of the most momentous judgments in human history when he wrote, “To this single idea must the revolution be ascribed, by which, after groping in the dark for so many centuries, natural science was at length conducted into the path of certain progress” [68].

Complexity trumps good fortune: the more complex the environment, the more the need for experimental design. This is evident in the words of Hans Reichenbach: “As long as we depend on the observation of occurrences not involving our assistance, the observable happenings are usually the product of so many factors that we cannot determine the contribution of each individual factor to the total result” [69]. Think of all the factors at work in a living cell.

Today we have a well-developed statistical theory of experimental design to assist the scientist in planning an experiment. Douglas Montgomery emphasizes the importance of design when he writes, “By the statistical design of experiments, we refer to the process of planning the experiment so that appropriate data will be collected, which may be analyzed by statistical methods resulting in valid and objective conclusions. The statistical approach to experimental design is necessary if we wish to draw meaningful conclusions from the data” [70]. All too often researchers collect data without a clear idea of what analysis is to be performed on the data, a form of stamp collecting as famously stated by Ernest Rutherford [71]; or planning to use methods that have never been justified for such data – or even been shown to be unjustified. Perhaps such groping in the dark may find gold when probing some simple humanly designed system, but the chances are slim to null in the hyper-complex environment of biology. 

The need for designed experiments places a double burden on the scientist, the need for mathematical knowledge with which to formulate potential relations to be embodied in a model and for biological knowledge with which to constrain the model space and articulate relevant experiments. As for what experiments to choose, here we depend on the intuition, one might even say cunning, of the scientist. The advancement and direction of science depends on choices. Erwin Schrodinger writes, “Consider the number of experiments which have actually furnished data on which the structure of physical science is based. That number is undoubtedly large. But it is infinitesimal when compared to the number of experiments that might have been carried out, but never actually have been. Therefore, a selection has been made in choosing the raw material on which the present structure of science is built.” [72]. A bit of meditation on the structure of the cell and one comes quickly to the conclusion that biologists had better exhibit quite a bit of cunning.

## HUMAN INTELLECTUAL CAPACITY

The requirements of the discovery process make it clear that, in addition to the limitations imposed by mathematical theory, computation, statistics, and experimentation, we are faced with fundamental limitations in terms of human intellectual capacity. The fact that there is a hard upper bound here can be seen by analogy. If we throw a ball to Maggie, she has outstanding ability to assess its trajectory, run swiftly to the area where she expects it to bounce, and then redirect her movement to catch the ball in her mouth before it hits the ground again. Her ability to swiftly gain knowledge is evidenced by how fast she learned all of this during the first initial tosses of the ball. But she cannot understand the differential equations to formalize these actions into a scientific theory. Her limitations in this direction are hardwired and she has no idea of what lies beyond that hardwiring. We are no different in this final respect, except that our limits lay farther out. We can understand the requisite differential equations but, like Maggie, we have no idea of what lies beyond our hardwiring. If there are categories beyond our hardwiring that are necessary for better scientific modeling, be it physics or biology, then we are both incapable of doing it and unaware of what it is that we cannot do.

Be that as it may, as a species we possess significant mathematical knowledge and therefore our practical problem is to put a good deal of that into our toolboxes. A scientist’s creative capacity is expanded by expanding his or her mathematical toolbox, as well his or her store of biological knowledge. Together, these form the knowledge base, which must be sufficiently large – that is, the base must contain the requisite knowledge required for a particular creative act. To formulate his theory of general relativity, Einstein had to learn Riemannian geometry. Had such a theory not existed, he would have been in the position of Newton, who had to invent the calculus to advance physical knowledge. Very few of us will ever make fundamental mathematical discoveries to advance science; the vast majority will have to be content with thinking in the framework of existing mathematical theories and, if necessary, like Einstein, expand our mathematical knowledge when necessary. There have been efforts in biological sciences to expand the mathematical framework [73, 74].

To think formally about biological networks, one must think in terms of mathematical categories that pertain to dynamical networks; otherwise, the thinking will be too limited in scope and depth. This limitation can only be overcome by greater knowledge, that is, by education. Absent a solid education, one cannot go beyond the superficial, especially in a subject such as biology (and medicine) that requires mathematical breadth in difficult areas like stochastic processes. Until this level of education is attained by working biologists, both biology and medicine will inevitably be limited because the human capacity for conceptualization will be lacking. 

When it comes to creative capacity in biology, we must recognize that we humans, who tend to think linearly, deterministically, and univariately, must step outside of our ordinary mental categories and think nonlinearly, stochastically, and multivariately. This is not easy. Our ordinary intuition fails. One sees this when researchers believe that the more measurements they make, the more knowledge they will gain. While such a conclusion may be a product of “common sense,” it can be quite wrong. 

For instance, consider the “peaking phenomenon” in classification, where, if one increases the number of measurements by measuring more features while keeping the number of measurements per feature constant, then it is commonplace for the performance of the designed classifier to first improve with more measurements and then degrade as more and more measurements are included in the design procedure [75, 76]. Fig. (**5**) illustrates the peaking phenomenon for classification [76]. It graphically displays the classifier error (vertical axis), which is the probability of an error when classifying any future observation, as a function of the sample size and the number of features (horizontal axes), when using linear discriminant analysis for classification in a particular Gaussian model. For a fixed sample size, the error first decreases as more features are used (meaning using more measurements) and then it begins to increase when the number of features passes some optimal value. It should be noted that this example is archetypal and the actual situation can be much more complicated [77].

Budding scientists must be wrenched from the categories of the everyday world and forced to think in the world of biological processes, a world very different from the one with which humans are familiar. As a society we need to recognize that there will be few who can make this transition of thought processes and that these must be identified and rigorously educated from an early age. This view was strongly espoused by A. N. Kolmogorov [78].

The full extent of this re-categorization of human thinking comes when we recognize that scientific knowledge is not synonymous with human intelligibility regarding Nature. This was appreciated by Newton, who recognized that his gravitational laws did not provide some kind of direct knowledge of Nature; rather, they described behavior. This limitation is natural because human sensation provides observations of phenomena, not of a natural order behind the phenomena. James Jeans puts the matter in practical terms:

- A mathematical formula can never tell us what a thing is, but only how it behaves; it can only specify an object through its properties. And these are unlikely to coincide *in toto* with the properties of any single macroscopic object of our everyday life…. We need no longer discuss whether light consists of particles or waves; we know all there is to be known about it if we have found a mathematical formula which accurately describes its behavior, and we can think of it as either particles or waves according to our mood and the convenience of the moment [79].

It is a form of naïve realism to believe that collecting a large library of facts about molecular interactions will lead to fundamental knowledge. Perhaps one may regard each fact as intelligible in its own right (although there certainly are epistemological issues with such an assumption), but one has no reason to expect that system behavior will be intelligible. Just as we have no experience with interactions at the quantum level, we have no experience with systems having hundreds of thousands of components interacting asynchronously, nonlinearly, multivariately, and stochastically. It would be naïve in the extreme to believe that the global behavior of such systems will be intelligible, that such systems can be represented outside the confines of complex mathematics, and that the behavior of such systems can be predicted absent the mathematical derivation of system characteristics. 

## CONCLUDING REMARKS

It is important to recognize limiting factors in the quest for knowledge because otherwise one might spend an inordinate amount of time and energy trying to solve a problem without realizing that one of these factors is blocking the way. An example of this conundrum occurs when one gets stuck trying to work with an overly complex mathematical model and fails to recognize that a reduced model will make the problem tractable. Another, in the opposite direction, is when one is stuck trying to model phenomena with an insufficient mathematical model owing to a lack of mathematical knowledge, for instance, trying to model an inherently stochastic process deterministically. Awareness of limitations facilitates efforts to push them back when necessary.

Here we come face to face with a different kind of human issue, one that is not technical, but one that is nonetheless very relevant to the progress of science: the desire for knowledge, or the lack thereof. As we have noted previously, it is rare for a scientist to develop a fundamentally new domain of mathematics, yet it is often the case that mathematical problems within an existing domain must be solved to advance science. A salient contemporary biological example concerns phenotype classification based on genomic features, in particular, gene expressions, where we are confronted with extremely large feature sets and small samples. Very little is known in regard to feature selection and error estimation in this environment, especially as to what conditions are required to facilitate good feature selection and error estimation [80, 81]. Nevertheless, rather than attack the root problems by obtaining the necessary mathematical and statistical knowledge for expanding our scientific knowledge, a host of papers has been published without the possibility of knowing whether the results are scientifically meaningful, in many cases, being virtually certain that they are not. 

To illustrate the point, consider error estimation via “cross-validation,” a method often employed in genomic classification. The basic idea is to first design a classifier by some method and then to estimate its error in the following manner: randomly partition the sample data into a collection of disjoint subsamples, for each subsample design a new “surrogate” classifier on the data not in the subsample, compute the numerical (counting) error of this classifier on the subsample, repeat the procedure for all subsamples, and then average the numerical errors of the surrogate classifiers to estimate the error of the originally designed classifier. The most basic form of cross-validation is to use subsamples of size one, so that the surrogate classifiers are all designed by leaving out a single data point, hence, the “leave-one-out” error estimate. Fig. (**6**) shows a scatter plot and linear regression line for the leave-one-out estimate (horizontal axis) and true error (vertical axis) [82]. What we observe is typical for small samples. Not only do we see large variance, just as strikingly we observe negligible regression between the true and estimated errors. Indeed, it has been mathematically shown that there can be negative correlation between the true and estimated errors in some basic models [83]. 

Earlier we discussed the danger of depending on common sense, in the sense that even in the presence of what seems like a reasonable mathematical method, rigorous proof is needed to establish the accuracy of the method. But with cross-validation, not only are we given no mathematical proof of its accuracy, where is the common sense? Is it intuitive to estimate classifier error rate on future observations by designing surrogate classifiers on portions of a small sample and averaging the errors they make on the portions left out? Some simple hand computations with pencil and paper will likely convince one otherwise. Nevertheless, in the absence of intuition or proof that cross-validation provides accurate estimates with small samples, and much evidence to the contrary [84, 85], a host of papers has used it and other related approaches. Should we be surprised that the results are not reproducible [86]? In fact, the performance of leave-one-out has been mathematically characterized for discrete classification [87] and linear discriminant analysis in a Gaussian model [88, 89] and it can be accurate when the true error is very small, but this requires the assumption of prior knowledge. Moreover, given the appropriate prior knowledge, the leave-one-out estimate can be calibrated to produce a better result [90]. But as typically used, on small samples without prior knowledge, cross-validation estimation is meaningless.

The issue here is one of purpose. Do we as a community sufficiently desire knowledge to address the difficult problems standing in the way of knowledge? There can be no doubt that human beings possess the intellectual capacity to solve many of the problems because, as a species, we have solved harder problems, so this is not an issue of human capacity; rather, it is an issue of human choice. 

In this regard, we close with some words of the Spanish philosopher Jose Ortega y Gasset: “Has any thought been given to the number of things that must remain active in men’s souls in order that there may still continue to be ‘men of science’ in real truth? Is it seriously thought that as long as there are dollars there will be science?” [91].

## Figures and Tables

**Fig. (1):**
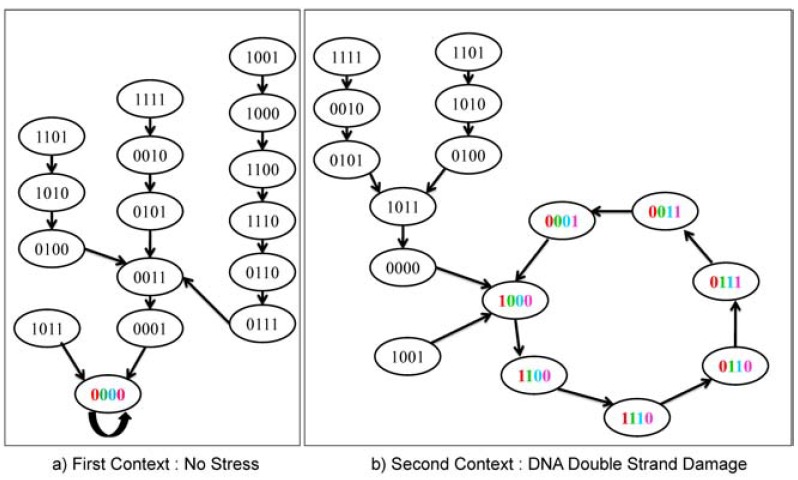
A network governing the response of p53 to DNA double strand breaks.

**Fig. (2):**
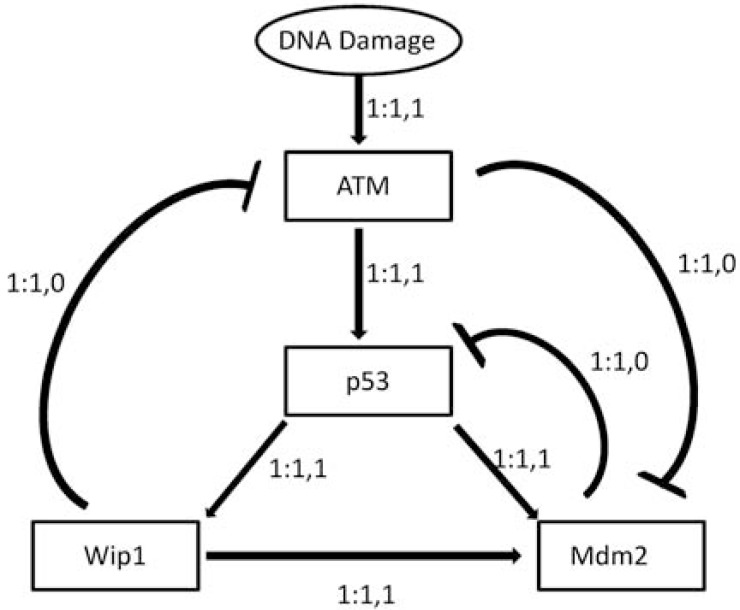
State space dynamics of Boolean networks corresponding to (**a**) no damage, dna_dsb = 0; and (**b**) double-strand-break damage, dna_dsb = 1.

**Fig. (3):**
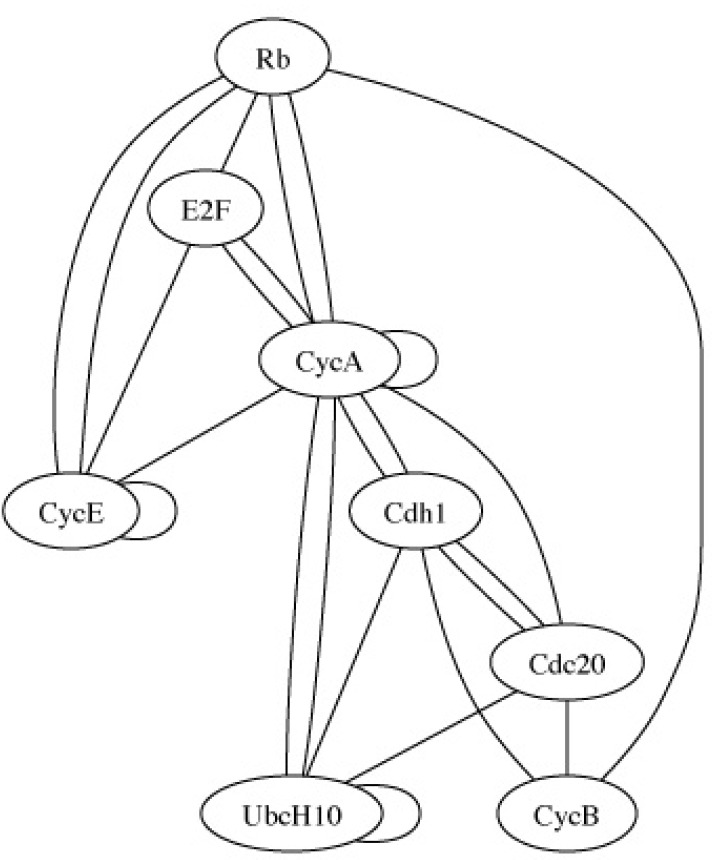
Connectivity graph for mutated Boolean cell cycle network.

**Fig. (4):**
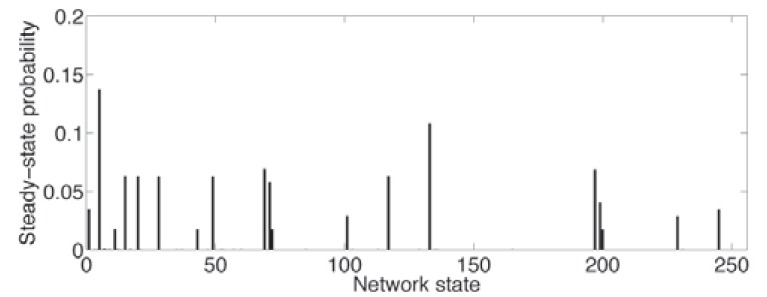
Steady-state distribution for mutated Boolean cell cycle network.

**Fig. (5):**
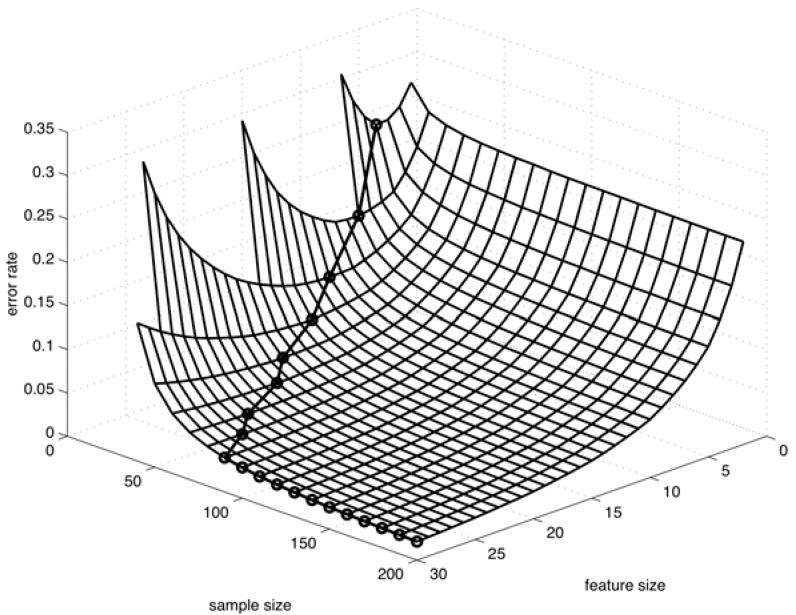
Peaking phenomenon.

**Fig. (6):**
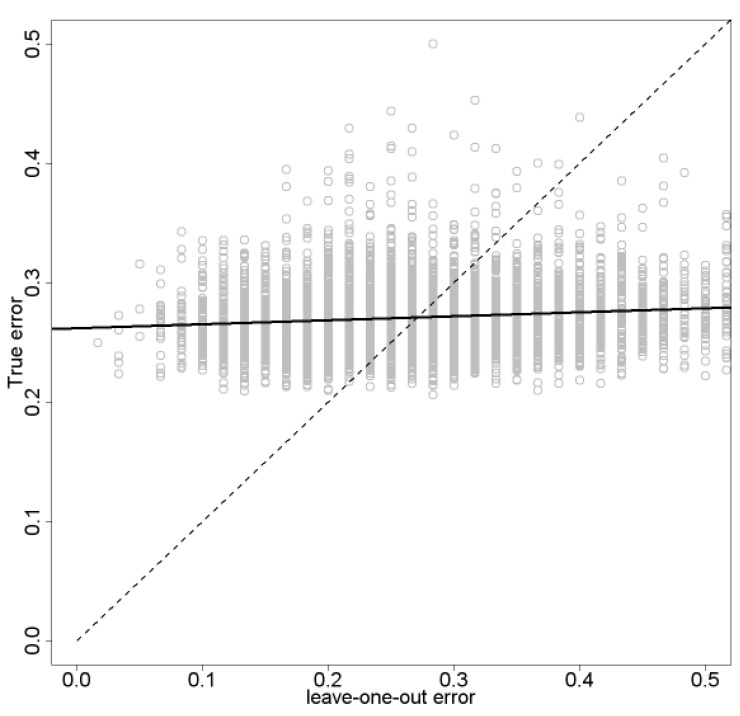
Scatter plot and regression line for leave-one-out error estimation.

**Table 1.** Logical Regulatory Functions for a Mutated Boolean Cell Cycle Network

Order	Gene	Regulating Function
*x*_1_	*CycD*	Extra-Cellular Signals
*x*_2_	*Rb*	$\bar{\mathrm{CycD}}\wedge \bar{\mathrm{CycE}}\wedge \bar{\mathrm{CycA}}\wedge \bar{\mathrm{CycB}}$
*x*_3_	*E2F*	$\bar{\mathrm{Rb}}\wedge \bar{\mathrm{CycA}}\wedge \bar{\mathrm{CycB}}$
*x*_4_	*CycE*	$\mathrm{E2F}\wedge \bar{\mathrm{Rb}}$
*x*_5_	*CycA*	$\left(\mathrm{E2F}\vee \mathrm{CycA}\right)\left(\bar{\mathrm{Rb}}\wedge \bar{\mathrm{Cdc20}}\wedge \bar{\mathrm{Cdh1}\wedge \mathrm{UbcH10}}\right)$
*x*_6_	*Cdc*20	*CycB*
*x*_7_	*Cdh*1	$\left(\bar{\mathrm{CycA}}\wedge \bar{\mathrm{CycB}}\right)\vee \mathrm{Cdc20}$
*x*_8_	*UbcH*10	$\bar{\mathrm{Cdh1}}\vee \left(\mathrm{Cdh1}\wedge \mathrm{UbcH10}\wedge \left(\mathrm{Cdc20}\vee \mathrm{CycA}\vee \mathrm{CycB}\right)\right)$
*x*_9_	*CycB*	$\bar{\mathrm{Cdc20}}\wedge \bar{\mathrm{Cdh1}}$
